# An international position paper on mother-infant (perinatal) mental health, with guidelines for clinical practice

**DOI:** 10.1007/s00737-016-0684-7

**Published:** 2016-11-08

**Authors:** Ian Brockington, Ruth Butterworth, Nine Glangeaud-Freudenthal

**Affiliations:** 1 University of Birmingham, Edgbaston, Birmingham, B15 2TT UK; 2 Inserm U 1153, Paris Descartes University, Paris, France

**Keywords:** Mother-infant psychiatry, Perinatal mental health, Clinical guidelines, Adjustment to pregnancy, The psychiatry of pregnancy, The psychopathology of parturition, Postpartum psychiatric disorders, Child-related maternal psychiatric disorders

## Abstract

The purpose of this paper is to set out informal, provisional and comprehensive but concise guidelines for mother-infant (perinatal) mental health (psychiatry), as an area of specialisation. It is *informal* in the sense that the authors are clinicians and researchers from many different nations, who share a common goal and vision, speaking on their own behalf and not with the backing of any authority or society. It is *provisional* in the expectation that it can be improved by criticism and new research findings. It is a *comprehensive* summary of the development of the specialty, its core knowledge and recommended investigations and interventions. It is *concise* (under 6,000 words, taking less than an hour to read) in order to increase readership and facilitate translation. No attempt has been made to parade the evidence for these suggestions, because the document would have been too long to translate, and for many to read. Instead, drafts were circulated for criticism by those included in the authorship, resulting in a consensus (finalised by the three principal authors), providing a framework to guide service provision, clinical practice and research. The full list of authors, from 33 nations, is given in the postscript. They include mother-infant (or parent-infant) and perinatal adult or child psychiatrists and those with a special interest; mother-infant, perinatal and forensic psychologists; psychiatric nurses; the founders of Postpartum Support International and the Association for Postnatal Illness; representatives of social work and obstetrics and the management of these services, and research scientists working in the field.

## Part 1

### The specialty of mother-infant psychiatry (perinatal mental health)

#### The need for this specialty

For most of those mothers in the world afflicted by psychiatric disorders related to childbearing, the only source of help and care is from non-specialised psychiatric services—either from adult or child psychiatry. It is our claim that the scale of the problem requires the development of its own specialty. This is for three reasons:

- The mental health services concerned with the pregnant and newly delivered mother have an equal responsibility for the child. These infants are vulnerable *inter alia* by dint of genetics, exposure to pharmaceutical agents, disrupted parental relationships and psychosocial risk factors. The child participates in a number of syndromes—in maternal anxiety about its health and survival, fear of the responsibility for child care, delusions or obsessions about the infant or disorders of the relationship with foetus and infant, especially emotional rejection and pathological anger. The unborn or newborn child has to be taken into account in many forms of treatment. The assessment of the mother’s relationship with the foetus and infant is a vital and specific part of the clinical investigation. These child-related features, together with observations of parent-baby interaction and the infant’s care and safety, are peculiar to this branch of psychiatry.
- Childbearing is attended by a plethora of psychiatric disorders, which make pregnancy and its aftermath the most complex event in human experience. The sheer number, variety and range of disorders tax the knowledge of general psychiatrists. Some are common and some are rare, although this may not be true in countries with high birth and maternal mortality rates, and rarity may merely reflect the lack of recent reports. Psychiatry has a duty to train and provide consultants with knowledge of the whole span of psychopathology, not just common disorders, and parents should have access to experts with comprehensive knowledge.
- Identifying parents and infants at risk during pregnancy and the postpartum period offers opportunities for the primary and secondary prevention of parental mental illness, and its adverse consequences on the development of children, resulting in long-term cost savings in health and other systems.

The specialty, with the knowledge and resources required to deal effectively with all these mental health disorders is usually called ‘perinatal psychiatry’, emphasising the birth and its effects on the parents. Some, however, prefer the term ‘mother-infant’, or related terms such as ‘parent-infant’ or ‘perinatal and infant’ psychiatry, which keep the baby in the title as an equally important focus. We have compromised by using both alternatives in the name of this specialty.

#### Historical development

This branch of psychiatry can trace its roots to the work of three French pioneers: Jean-Étienne-Dominique Esquirol (Esquirol 1819) published the first detailed asylum survey among women admitted after childbirth to the Salpêtrière Hospital in Paris. Louis-Victor Marcé (Marcé 1858) published the first monograph covering the whole field as then known. Ambroise Tardieu (Tardieu 1860) drew attention to child maltreatment, reporting a large series and describing the effects on the children. In England, a pioneering initiative was taken in 1948 by Tom Main (Main 1958), who negotiated the introduction of a toddler into the Cassel Hospital in Middlesex; as a psychoanalyst, he emphasised the opportunity offered to study the mother-child relationship. Independently, Gwen Douglas (Douglas 1956) reported the admission of six mothers and infants to the West Middlesex Hospital; her first patient also had a severe mother-infant relationship disorder. About the same time, Paul-Claude Racamier in France (Racamier 1961) and James Haycock and Henry Grunebaum (Grunebaum and Weiss 1963) in Massachusetts opened units for conjoint in-patient care, and these initiatives were the first step in the development of mother and baby units in Britain, France, the USA, Australia, Belgium, the Netherlands, Germany, Canada, New Zealand, Israel and India (and possibly other countries). By providing a focus for the referral of many mothers, it has led to the recognition of a raft of new disorders, with ideas for therapy and the development of therapeutic teams. In some countries, these services have developed with a link to child psychiatry, and in others, to adult psychiatry or both. Services dealing with the mental health of fathers are also developing.

#### Worldwide distribution

We have sought information about specialised services for mothers and infants in all nations with at least five million population and more than twice the mean world gross domestic product (GDP) per head; it seemed reasonable to expect that they would have made some provision for mothers and infants, as have been made by some nations with a lower GDP per head (for example Brazil, India, Mexico and Turkey). It was difficult to obtain this information: our account will contain errors and we welcome their correction.

No nation has come near to meeting the needs of mothers and their infants; where specialist services exist, about 5 % of recently delivered mothers gain access—many others do not. An assessment made in Birmingham, which had day care and a dedicated community service, indicated a requirement of at least five in-patient mother and baby places/million population. So far as we have been able to ascertain, the nation with the most complete spread of services is Australia, which has in-patient units in all major cities, although many of these are private and not accessible to all; but even in Melbourne (serving the State of Victoria, population six million), which has 30 publicly funded beds (the only place in the world with the recommended bed level) together with residential early parenting services admitting 1000 mothers/year, there is pressure on the service. In Britain, where generous funding has been provided by the National Health Service, less than half the number of required beds has been achieved; France, New Zealand and Switzerland have a similar number. Austria, Belgium, Germany, Israel, Japan and the Netherlands have bed places but less than one/million population. The USA has admitted mothers and infants in the past, but at present has only three out-patient units. Hong Kong, Iceland, Italy, Norway, Singapore, Spain and Taiwan have out-patient services dedicated to mothers and infants. It appears that Denmark, Eire and Sweden have no specialist services. Thus the service is inadequate, with regional disparities, even in the pioneering nations. In many, funding is precarious. Nonetheless, in India, with a mean GDP/head just over one third of the world average, it has been demonstrated in Bangalore that conjoint admission can be achieved. An initiative, therefore, first taken over 60 years ago, which offers unusual opportunities for research and prevention of mental disorders in the mother and child, is still in the early stages of development.

#### The many styles and settings for the practice of this discipline

The essence of this specialty is its core knowledge, not exclusively a multi-disciplinary team or an expensive resource. There are also scientists, whose research makes a valuable contribution, and those with responsibility for public health, finance and service planning, who have no personal clinical involvement. It can be practised from an office, on an out-patient basis by a professional of any discipline working alone, who has acquired this knowledge and applies it in clinical practice. It can be practised by consultation-liaison psychiatrists or psychologists, or those with other responsibilities and a part-time interest. Primary care and community health workers make a major contribution to the care of mentally ill parents and their infants, especially in countries with less well-developed psychiatric services.

##### Day units

This specialty can be practised with great effect from day hospitals as in many French, Belgian and German units and in Hanley, UK; this is less disruptive and much less costly than in-patient admission, although there are limits imposed by geographic distance. In the right circumstances, day units can deal with all but the most severe and dangerous disorders and provide mothers with an opportunity to meet others with similar problems.

##### Conjoint in-patient care

The full panoply of resources includes an in-patient unit for mother and baby (conjoint) admission. To ensure the safety of the infant, a high staff-to-mother ratio is essential, with costs as high as any psychiatric units. In India, relatives have been recruited to care for mother and baby. Facilities shared with acute psychiatric wards may be a compromise for isolated towns, although there are concerns about the safety of the infants. Some units admit infants only below the age of 6 months, some up to the age of 2 years or more. Some can offer apartments to admit whole families. Some admit pregnant women. Some are only open during the working week. Some share facilities with other disorders, such as anorexia nervosa. Some are based in obstetric or paediatric units. Unfortunately, no high-income nation has yet established the cost effectiveness of these expensive units by rigorous comparison with non-specialist care, nor properly assessed their safety.

##### Community outreach

It is good practice, if possible, to provide a specialist community service, collaborating with day- or in-patient units. This minimises the disruption to family life; it is difficult for a parent with a baby or young family to travel to a hospital for consultation. There is a special quality to assessments made in the home. Exceptionally, and with due precautions, it has been possible to treat severe disorders at home. The deployment of community nurses for treatment and home follow-up is ideal, integrating with community services and primary care. In services with responsibility for a large area (such as that provided from Christchurch for the South Island in New Zealand), there is a need to travel regularly to distant locations for teaching and clinical supervision, or to hold video-conferences.

#### Functions of the service

The scope of the specialty is wide. It involves prepartum as well as postpartum disorders and, on the fringes, the rare complications of abortion, weaning and adoption. Its functions include the following:

- Accurate diagnosis. This depends on the acumen of the clinicians and their grasp of the full range of disorders in this most complex area of psychiatry.
- Treatment. A specialised team can serve as a referral centre for a wide area, serving a population of several millions, with more than 10,000 births/year. It can advise local practitioners and general psychiatrists and take over the management of severe and intractable disorders.
- The training of staff from several disciplines, including medical and nursing students, psychiatrists, nurses, midwives, psychologists and social workers, about diagnosis, therapy, prevention and risk assessment.
- The development of services in its locality, advice on national development and public and family education to improve awareness and reduce stigma, mobilising support for funding.
- Research into causation, treatment and prevention, including clinical observation.
- A strong working relationship
  • with social services and child protection agencies,
  • with obstetric and neonatology services,
  • with paediatricians, lactation consultants, general practitioners and other members of primary health teams and
  • with the voluntary sector that provides much support to mothers in difficulty; it is useful to keep a register of consenting mothers as another source of mutual support.
- Medico-legal work, supporting the interests of the mothers and infants in its care, working with child protection agencies in residence and custody disputes, advising in cases of child maltreatment and infanticide and supporting mothers who suffer intimate partner violence.

#### The multi-disciplinary team

These services benefit from the collaboration of different professions; they can best be practised by a dedicated multi-disciplinary team, whose members have read widely and are up-to-date in a rapidly developing discipline. The team should ideally include the following, either as members or available for consultation:

- Psychiatrists with appropriate training.
- Clinical psychologists, who can deploy and supervise a number of specific therapies.
- Trained mental health case managers.
- Nursing staff of several kinds—enough in-patient nurses to provide care round the clock and throughout the week, nursery nurses to care for the infants, mother-craft nurses, and public health and community nurses who can treat mothers at home.
- Midwives working in antenatal clinics and postnatal wards and in a public health role; in Africa, traditional birth attendants have a similar role.
- Psychotherapists of various kinds and disciplines.
- Social workers not only to conduct social casework with families but also to manage the relationship with social and child protection agencies in the locality served.
- Occupational and art therapists, who have a role in psychotherapy, rehabilitation and habilitation in mothering skills.
- Paediatricians for consultation on neonatal health.

## Part 2

### The knowledge base

#### The disorders

These can be placed under five headings—adjustment to pregnancy, psychiatric complications of pregnancy, the psychopathology of parturition, postpartum mental illness and disorders involving the child.

#### Disorders of adjustment to pregnancy

Conception can be a crisis. It has been estimated that about half of pregnancies are unplanned and, although many were merely mistimed and most are well accepted, unwanted pregnancy is a problem in many nations and countries of various religious faiths. The psychiatric complications of termination of pregnancy are controversial, but abortion (whether spontaneous, medical or social) is occasionally followed by psychosis (organic, psychogenic or bipolar). There is a large literature on denial of pregnancy. About 10 % of pregnancies are rejected, with an increased risk of postpartum disorders including emotional rejection of the child. There is a pathology of the mother-foetus relationship which, in those rejecting their pregnancy, can lead to foetal abuse.

#### Other psychiatric complications of pregnancy

Depression is frequent and just as common during pregnancy as after it or at other times in women of this age group; it may be recurrent with each pregnancy. In many cases, it is related to social isolation and stress, such as friction with spouses or partners, especially those that are critical, controlling, coercive or abusive; intimate partner violence can increase during pregnancy. Anxiety is common, with the main focus on the fear of foetal loss (especially in those with a history of infertility, *in vitro* fertilisation, miscarriage or stillbirth), of lack of support, of inadequacy as a mother, or of parturition (tocophobia). There is an increase in obsessive-compulsive disorders. Conjugal jealousy may be awakened by the disturbance of intimate life.

Although less frequent than after the birth, many forms of psychosis occur during pregnancy: among the organic psychoses are episodes complicating chorea gravidarum, and the Wernicke-Korsakow syndrome complicating pernicious vomiting, as a result of rehydration without thiamine treatment. Non-organic psychoses are heterogeneous: some are sporadic, some are chronic disorders that began before conception or recur because prophylaxis was stopped and some are bipolar/cycloid disorders.

Eating disorders may cause difficulties in pregnancy, especially if the mother is actively restricting her diet. Some women develop a form of dysmorphophobia for their pregnant appearance, which may include ideas of reference and social avoidance. Occasionally, factitious disorders (deliberately feigned symptoms) present during pregnancy. While some women long for the end of pregnancy, a few importunately demand premature delivery at the risk of the baby’s health. Drug abuse during pregnancy is a major problem, with serious risks for the child, requiring skilled management in collaboration with obstetricians and medical and social community services.

#### The psychopathology of parturition

In many parts of the world, childbirth takes place without skilled assistance and without adequate analgesia. Where maternal mortality is high, there are several complications (for example, delirium and stupor), which are now rare in high-income nations. Other organic psychoses may occur during parturition, including eclamptic and epileptic psychoses. Bipolar psychoses may start during labour. At the height of the pain, rage and acts of desperation have been reported, including suicide and auto-Caesarean section. Where the pregnancy was concealed, neonaticide was at one time the commonest form of murder. Even with modern obstetrics and analgesics, labour can still be a severe ordeal, and childbirth may reactivate past trauma. Post-traumatic stress disorder, with intrusive flashbacks, nightmares, phobic avoidance of reminders and a prolonged high tension state, has been reported in up to 5 % of births. Another response to traumatic labour is a complaining reaction, with an intense preoccupation with claimed malpractice, which may go beyond litigation to fantasies of revenge. Traumatic birth may also affect bonding.

#### Postpartum psychiatric disorders

##### Depression

This is much researched. At the level of referral to specialist services, it occurs after about 5 % of births (though much higher in community surveys). It is probably not more common than in other women in this age group. It tends to be a ‘catch-all’ term covering a complex variety of distinct disorders; some mothers with ‘postnatal depression’ suffer from other disorders, and, in most, there is more than one disorder present. Nevertheless, postpartum depression is a major source of referral. It is important to investigate its causes; some cases are a manifestation of bipolar disorder, but others are associated with a wide variety of psychosocial stresses, including difficulties in infant care. The treatment of the mood disorder and its determinants is a large part of the work of mother-infant (perinatal) services in many countries.

##### Anxiety and related disorders

Research on postpartum anxiety is somewhat neglected: its focus is often child-related (see below), but fear of criticism of childcare and lack of support are also common. Childbirth is among the main triggers of obsessive-compulsive disorders, which may include a worsening of longstanding obsessional traits, or new onsets of obsessive cleaning or housework, as well as child-related obsessive ideas. As in pregnancy, dysmorphophobic ideas about weight gain, stretch marks or breast size may become symptomatic.

##### Psychoses

Postpartum psychoses are of many forms and causes. There are many organic psychoses, and most occur shortly after the birth. Historically (and perhaps still in some parts of the world), infective delirium and eclamptic psychoses rival the frequency of non-organic psychoses. Delirium and stupor (similar to that which occurs during delivery) may occur immediately after the birth and may be recurrent. Cerebral venous thrombosis is common in India, and other rare causes include hypopituitarism, water intoxication, withdrawal of ethanol or other ‘substances’, hyperammonaemia and epilepsy.

Psychoses that start within the first 2 weeks (which are the majority) in high-income nations are usually bipolar/cycloid disorders; they may also begin 4–13 weeks after delivery (perhaps at the first postpartum menses) and after weaning. Although a majority of non-organic psychoses are manic-depressive, others are schizophrenic, depressive, paranoid or psychogenic. Postpartum bipolar/cycloid disorders notoriously recur, with an increased risk after future pregnancies, but are also relapsing (that is, recur within a few weeks), and there is evidence that these relapses are related to menstruation. Bromocriptine, given to stop lactation, can also provoke a psychosis.

#### Child-related disorders

The infant modifies maternal psychopathology in many ways. Infant-centred anxiety takes two contrasting forms: some mothers, especially first-time mothers, are terrified of the overwhelming responsibility for childcare; in extreme cases, this can lead to puerperal panic and phobic avoidance of the newborn. More common is a morbid enhancement of the concern, which all mothers have, for the safety and health of their child. A mother with a pathological fear of cot death resists sleep because of the need to monitor the baby’s breathing, resulting in exhausting sleep deprivation. Among the obsessional disorders is the obsession of infanticide, where a gentle and devoted mother is besieged by fantastic images of destroying her child; these impulses must be distinguished from anger-based impulses to abuse a child, experienced by mothers with impaired bonding. The delusions of psychotic mothers often involve the child and may be dangerous. Various maternal preoccupations, such as conflicts resulting from her own history or from recent traumatic experiences, can impair childcare and sensitivity to infant cues.

A proportion of mothers take time to bond, but a small minority may suffer from emotional rejection of the infant, which, together with psychosis and suicidal depression, is in the first rank of severity in this area of psychiatry. Its causes are not well understood, but probably include the mother’s own experience of trauma, abuse and loss (with the danger of perpetuating an intergenerational cycle of abuse), as well as rejected pregnancy and depression. Its long-term effects, which include child maltreatment, are the most severe of all postpartum psychiatric disorders. It is not part of the syndrome of ‘postnatal depression’—some of these mothers are not depressed, and, when they are, the severity and course of the two disorders differ; their treatments and to some extent their causes are different.

Some extreme risks to parents and child must be considered—child maltreatment (by physical abuse, neglect and emotional abuse), suicide and filicide, which may be part of suicidal activity. Longstanding aggressive and antisocial traits, and other evidence of personality disorder, must be identified and taken into account when considering the infant’s safety. It is essential to take time to assess these risks and look for solutions. A cautious approach is recommended, and may involve close supervision, or, with careful preparation, separating parent(s) and child in the short or long term.

## Part 3

### Investigation and intervention

#### Pre-conception planning

Where possible, women with known mental illness, particularly those taking prophylactic medication, should be offered pre-conception counselling regarding the risks of pregnancy (to themselves and the child) and the risk and benefits of various treatment options during pregnancy and breast-feeding.

#### Screening

Attendance at antenatal clinics is a first-class opportunity for finding pregnant women at high risk, such as those with a history of depression or psychosis, drug or alcohol abuse, major social problems or unwanted pregnancy. After the birth, maternity staff can make close observations of the initial responses of vulnerable mothers, and in many countries, staff from various disciplines visit the mother at home. Those involved in screening should know how and to whom they can refer if difficulties are identified, as no screening is effective without a supportive network for intervention.

A range of screening questions or questionnaires may be used to determine which mothers may need referral for further diagnosis and treatment. It is best to begin with general, open questions rather than specific enquiries about depression or anxieties. For example, one could ask,

“How do you feel about this pregnancy?”

“What are your main worries at the present time?”

“How are things going with (name of baby)?”

Each professional will have his or her favourite questions. Observations of the mother’s appearance or handling of the baby are equally important. The Edinburgh Postnatal Depression Scale (Cox et al. 1987) has been widely evaluated and used and translated into many languages. If there are difficulties in a mother’s emotional response to pregnancy or the baby, there are several useful scales: during pregnancy, there is the Prenatal Attachment Inventory (Müller 1993). After the birth, there is the Postpartum Bonding Questionnaire (Brockington et al. 2006), which has been validated and widely translated.

#### Planning during pregnancy

If a mother with a severe mental disorder becomes pregnant, a multi-disciplinary planning meeting should be convened as soon as possible, to share information and coordinate management. The reason for urgency is that the interval between diagnosis of pregnancy (which may be delayed) and birth (with may be premature) can be short. The meeting should include all those involved in treatment, which will vary from country to country: the full list includes the general practitioner, a representative of the obstetric and mental health teams, (if appropriate) a social worker and (if possible) the expectant mother and family members. There are many issues to be addressed—pharmaceutical treatment, antenatal care, early signs of a recurrence, the management of the puerperium, the care of the infant and sometimes action to protect the child. It is important that the mental health team be alerted as soon as the mother goes into labour.

#### Interviewing

Once a mother has been referred to the service, in addition to the standard psychiatric history, it is essential to explore thoroughly the current pregnancy and birth.

During pregnancy, the interview should cover:

- The pregnancy’s social, cultural, psychological, psychiatric and obstetric background
- The circumstances under which conception occurred
- The expectant mother’s reaction and adjustment to the pregnancy, and her expectations of maternity
- The reaction of others, especially the baby’s father
- Changes in life-style including the mother’s sacrifices to complete this pregnancy
- Her burgeoning relationship with the unborn child
- Her health (mental and physical) during pregnancy
- Her worries and preoccupations

Next comes parturition and its effect on mother and neonate.

After the birth, the interview should cover:

- The mother’s reaction to the new-born and to infant feeding and care
- The father’s reaction and participation in infant care
- The effect of the birth on the family circle
- The support available to the young family
- Sleep deprivation and medical complications
- Psychiatric disorders including anxiety, morbid preoccupations, irritability, depression and psychotic symptoms

Finally, there is the mother-infant relationship:

- As background, the baby’s health, temperament, development and any specific difficulties
- The timing and quality of the mother’s emotional response
- Evidence of pathological anger and rejection
- The safety of the infant with these parents

There may be other interviews, but all these areas are covered in the Stafford Interview (the sixth edition of the Birmingham Interview), developed over the course of 22 years and translated into several languages (Brockington et al. 2016). It is useful in training, clinical practice and research.

#### Wider investigations

Where there is a possibility of an organic psychosis, physical examination, laboratory tests, electro-encephalograms and brain scans may be diagnostic. The hormonal status can be used to monitor the menstrual cycle.

#### Observations in day-patients and in-patients

In some mothers, day-to-day nursing observation of infant care and the mother-infant relationship are possible. There are rating scales such as the CARE Index, but the most important is narrative description:

- Recording the mother’s statements about the infant
- Noting her competence, skill and attunement
- Observing her response at times of separation and meeting, when the baby cries and makes demands, at feeding and bathing, and at times of crisis
- Observing the baby’s social behaviour independently of the parents, and comparing it with norms for that age and with the mother’s own perception of it
- Assessing the baby’s physical and psychological safety.

#### Therapy

A great deal can be done, using the full range of psychiatric and psychological treatments, to improve the mental health of pregnant and newly delivered mothers, their partners and infants. The costs and benefits of treatment options for parents and child should be discussed with all the participants—the mother and her wider family, the mental health team and the obstetric service. This requires a compassionate approach and a strong therapeutic alliance. The history of treatment compliance will also influence the balance of the cost-benefit analysis.

Therapy in pregnancy and the postpartum period is a rapidly developing field in terms of both pharmaceutical and psychotherapeutic interventions. We, therefore, hesitate to make any dogmatic statements and instead recommend that all clinicians maintain an up-to-date knowledge of the emerging evidence-base and local recommendations. The following sections outline some of the more solid conclusions from the available evidence.

##### Pharmaceutical treatment

During pregnancy, the embryo and foetus influence pharmaceutical treatment, and all drugs must be carefully monitored, and prescribed or withdrawn after a cost/benefit analysis for this pregnant woman. Certain drugs are teratogenic in the early stages, especially valproate (spina bifida). Antidepressant agents can lead to mild neonatal withdrawal symptoms. Given during parturition, benzodiazepines may intoxicate the infant. Occasionally, neuroleptics have caused side effects in the newborn.

Mood stabilisers, such as lithium and carbamazepine, given during pregnancy, reduce the recurrence rate in bipolar disorder. Parturition alters lithium clearance, and there have been a number of reports of dangerous levels of lithium in mothers taking their normal dose.

ECT can be given during pregnancy, but there are complications including preterm labour, which can be suppressed by tocolytic drugs such as terbutaline.

In the postpartum period, lithium, given immediately after the birth, can reduce bipolar episodes. Mothers with bipolar/cycloid disorders are highly susceptible to side effects from neuroleptics, and there have been several cases of neuroleptic malignant syndrome. The effects on breast-fed infants are minor, and even lithium has only occasionally led to adverse effects; but fever, gastro-intestinal illness and electrolyte loss could result in toxicity and this is a concern in nations where dysentery rates are high.

##### Psychological treatment

This is important in almost all cases, with many different approaches. It may be preventive, for example, preparing a tocophobic mother for the birth. It may involve antenatal education or focus groups to promote understanding of the psychology of parenthood.

Crisis intervention may be required, for example, in obstetric and paediatric consultations.

Specific psychological treatments are available for prepartum and postpartum anxiety, obsessional disorders, post-traumatic stress disorder and complaining disorders.

Interpersonal psychotherapy focuses on the resolution of emotional conflicts. Cognitive therapy is directed at the correction of maladaptive thoughts and beliefs. Group therapy, psychoanalysis, mindfulness and yoga are available in some services.

In major disorders of the mother-infant relationship, depression or other disorders should be identified and thoroughly treated as a first step. In addition, a wide variety of interactive therapies have been developed, such as baby massage, parent-infant psychotherapy (including play therapy and Wait, Watch & Wonder) and video interaction techniques. Occasionally, the parents may decide to relinquish the baby, and this decision must be respected and supported throughout.

Working with fathers (if possible) and involving them in the therapeutic process is important in the development of this specialty. They may need personal support. In some cases, couple therapy or family therapy is indicated.

## Conclusions

Once again, we emphasise that this is a provisional document, based on expert consensus. We invite and welcome critical comment, with the limitation that the document must be concise—readable and translatable.
